# The influence of marital status on the stage at diagnosis, treatment, and survival of adult patients with gastric cancer: a population-based study

**DOI:** 10.18632/oncotarget.7399

**Published:** 2016-02-15

**Authors:** Jieyun Zhang, Lu Gan, Zhenhua Wu, Shican Yan, Xiyu Liu, Weijian Guo

**Affiliations:** ^1^ Department of Medical Oncology, Fudan University Shanghai Cancer Center, Shanghai 200032, P.R. China; ^2^ Department of Breast Surgery, Fudan University Shanghai Cancer Center, Shanghai 200032, P.R. China; ^3^ Department of Oncology, Shanghai Medical College, Fudan University, Shanghai 200032, P.R. China; ^4^ Department of Surgery, Shanghai Medical College, Fudan University, Shanghai 200032, P.R. China

**Keywords:** gastric cancer, marital status, SEER, survival analysis, subgroup analysis

## Abstract

**Background & Aims:**

Marital status was reported as a prognostic factor in many cancers. However, its role in gastric cancer (GC) hasn't been thoroughly explored. In this study, we aimed to investigate the effect of marital status on survival, stage, treatment, and survival in subgroups.

**Methods:**

We used the Surveillance, Epidemiology and End Results (SEER) database and identified 16910 GC patients. These patients were categorized into married (58.44%) and unmarred (41.56%) groups. Pearson chi-square, Wilcoxon-Mann-Whitney, Log-rank, multivariate Cox regression, univariate and multivariate binomial or multinomial logistic regression analysis were used in our analysis. Subgroup analyses of married versus unmarried patients were summarized in a forest plot.

**Results:**

Married patients had better 5-year overall survival (OS) (32.09% VS 24.61%, P<0.001) and 5-year cancer-caused special survival (CSS) (37.74% VS 32.79%, P<0.001) than unmarried ones. Then we studied several underlying mechanisms. Firstly, married patients weren't in earlier stage at diagnosis (P=0.159). Secondly, married patients were more likely to receive surgery (P < 0.001) or radiotherapy (P < 0.001) compared with the unmarried. Thirdly, in subgroup analyses, married patients still had survival advantage in subgroups with stage II-IV and no radiotherapy.

**Conclusions:**

These results showed that marital status was an independently prognostic factor for both OS and CSS in GC patients. Undertreatment and lack of social support in unmarried patients were potential explanations. With the knowledge of heterogeneous effects of marriage in subgroups, we can target unmarried patients with better social support, especially who are diagnosed at late stage and undergo no treatment.

## INTRODUCTION

More than 990 million people are diagnosed with gastric cancer (GC) per year and 738 million of them die from this kind of cancer specifically.[1] Less than a century ago, GC was the most common cancer in the US. GC always leads to poor survival because of insensitivity and early resistance to chemotherapy.[2] Although the incidence rate and mortality of GC have obviously declined owing to the treatment of H. pylori infection, GC remains the most prevalent cancer in Asia and the second most common cause of cancer death globally.[1, 3–5]

Social support provides survival advantages to patients for many important causes of death.[6] Martial status is often regarded as the most important type of social support, since it is relevant to a great many physiological mechanisms affecting survival and associated with a variety of other important social support.[6, 7] Recently, many researches indicated that marital status is an independent prognostic factor of many kinds of cancers.[8–12] In previous studies, the effect of marriage on GC remained controversial. Marital status was reported as a good prognostic factor for survival of patients with GC by Kravdal et al. and Goodwin.[9, 13] On the contrary, Zare et al. reported that the survival rate of married patients with GC was lower than the singles.[14] However, These studies always take overall survival (OS) into consideration while neglecting gastric cancer-caused special survival (CSS). Meanwhile, social network and socioeconomic factors in US have remarkably changed as decades passed. To our knowledge, no research so far has shown the detailed methods by which marital status effects OS and CSS of GC. Several factors influenced by marital status, Including delayed diagnosis, no treatment and lack of social support, was supposed to lead to poor survival.[13, 15, 16] Hence, it is important to explore the underlying mechanisms of the relationship between marital status and GC.

In this study, we used data in 2004-2012 from the US Surveillance, Epidemiology and End Results (SEER) cancer-registry program that covers 30% of US population to explore the correlations between marital status and survival in patient with GC in US. We hypothesized that marital status probably affected survival of GC patients from aspects of stage at diagnosis or post-diagnosis factors including the choice of treatment and social support.

## RESULTS

### Clinicopathological baseline characteristics

According to the inclusion criteria, we finally enrolled 16910 eligible GC patients in our study. Of these, 7028 patients (41.56%) were married and 9882 patients (58.44%) were unmarried. Unmarried group included single, divorced/separated, and widowed groups, among which there was no difference of CSS in univariate log-rank test (P= 0.1626) (Supplementary Figure S1), hence we put them in the same class as unmarried group. There were significant differences in clinicopathological characteristics including sex, race, age, histotype, primary site, TNM stage, cause of death, grade, selection of surgery, and selection of radiotherapy between married and unmarried groups. Compared with unmarried patients, patients in the married group were more likely to be male and white. Age of married patients had a better chance to be in groups of 28-37, 38-57, and 58-69. Patients in the married group had more tumors at stage II, stage III, stage IV, grade III and grade IV. Married patients also had a larger proportion as alive or dead of other caused. More surgeries were performed on married groups including non-total or non-near-total gastrectomy, and total or near total gastrectomy. It was the same with radiotherapy. The demographics, clinicopathological characteristics of tumors and treatment types with different marital statuses were summarized in Table 1.

**Table 1:** Characteristics of patients by marital status, unmarried^a^ verus married. SEER 2004-2012 (n=16910)^b^

Characteristics	Total	Unmarried	Married	P value^c^
	16910 (100)	7028 (41.56)	9882 (58.44)	
**Sex**				<0.001
male	9463 (55.96)	2917 (41.51)	6546 (66.24)	
female	7447 (44.04)	4111 (58.49)	3336 (33.76)	
**Race**				<0.001
white	10298 (60.9)	4155 (59.12)	6143 (62.16)	
black	2804 (16.58)	1673 (23.8)	1131 (11.45)	
American Indian or Alaska Native	159 (0.94)	73 (1.04)	86 (0.87)	
Asian or Pacific Islander	3593 (21.25)	1111 (15.81)	2482 (25.12)	
Unknown	56 (0.33)	16 (0.23)	40 (0.4)	
**Age**				<0.001
18-27	84 (0.5)	56 (0.8)	28 (0.28)	
28-37	437 (2.58)	179 (2.55)	258 (2.61)	
38-57	3877 (22.93)	1379 (19.62)	2498 (25.28)	
58-69	4442 (26.27)	1549 (22.04)	2893 (29.28)	
70-84	6376 (37.71)	2770 (39.41)	3606 (36.49)	
85+	1694 (10.02)	1095 (15.58)	599 (6.06)	
**Histotype**				<0.001
Adenocarcinoma, NOS	8279 (48.96)	3561 (50.67)	4718 (47.74)	
Adenocarcinoma, intestinal type	2435 (14.4)	1014 (14.43)	1421 (14.38)	
Carcinoma, diffuse type	1084 (6.41)	429 (6.1)	655 (6.63)	
Tubular adenocarcinoma	166 (0.98)	59 (0.84)	107 (1.08)	
Papillary adenocarcinoma, NOS	41 (0.24)	18 (0.26)	23 (0.23)	
Mucinous adenocarcinoma	311 (1.84)	147 (2.09)	164 (1.66)	
Signet ring cell carcinoma	4594 (27.17)	1800 (25.61)	2794 (28.27)	
**Site**				0.026
Fundus of stomach	857 (5.07)	343 (4.88)	514 (5.2)	
Body of stomach	2115 (12.51)	875 (12.45)	1240 (12.55)	
Gastric antrum	5440 (32.17)	2295 (32.66)	3145 (31.83)	
Pylorus	839 (4.96)	377 (5.36)	462 (4.68)	
Lesser curvature of stomach, NOS	2207 (13.05)	891 (12.68)	1316 (13.32)	
Greater curvature of stomach, NOS	990 (5.85)	370 (5.26)	620 (6.27)	
Overlapping lesion of stomach	1892 (11.19)	777 (11.06)	1115 (11.28)	
Stomach, NOS	2570 (15.2)	1100 (15.65)	1470 (14.88)	
**TNM Stage^d^**				<0.001
Stage I	4468 (26.42)	1995 (28.39)	2473 (25.03)	
Stage II	3724 (22.02)	1537 (21.87)	2187 (22.13)	
Stage III	4077 (24.11)	1613 (22.95)	2464 (24.93)	
Stage IV	4641 (27.45)	1883 (26.79)	2758 (27.91)	
**Cause of Death**				<0.001
Alive or dead of other cause	7811 (46.19)	3068 (43.65)	4743 (48)	
Dead (attributable to this cancer)	9099 (53.81)	3960 (56.35)	5139 (52)	
**Grade**				<0.001
Grade I (well differentiated)	598 (3.54)	265 (3.77)	333 (3.37)	
Grade II (moderately differentiated)	3696 (21.86)	1604 (22.82)	2092 (21.17)	
Grade III ( poorly differentiated)	10534 (62.29)	4235 (60.26)	6299 (63.74)	
Grade IV (undifferentiated)	340 (2.01)	133 (1.89)	207 (2.09)	
Cell type not determined	1742 (10.3)	791 (11.25)	951 (9.62)	
**Surgery**				<0.001
No surgery	5438 (32.16)	2572 (36.6)	2866 (29)	
Non-Total or Non-near-total gastrectomy	8953 (52.95)	3540 (50.37)	5413 (54.78)	
Total or near total gastrectomy	2519 (14.9)	916 (13.03)	1603 (16.22)	
**Radiotherapy**				<0.001
No radiotherapy	12789 (75.63)	5591 (79.55)	7198 (72.84)	
Radiotherapy	3868 (22.87)	1337 (19.02)	2531 (25.61)	
Radiotherapy unknown	253 (1.5)	100 (1.42)	153 (1.55)	

Abbreviation: NOS= no other specific; SEER=Surveillance, Epidemiology and End Results; IQR=interquartile range.

^a^Including divorced, separated, single (never married), and widowed

^b^Data are presented as No.(percentage) of patients.

^c^P value of the Chi-square test or Wilcoxon-Mann-Whitney test comparing unmarried and married groups

^d^Being restaged according to the criteria of AJCC Cancer Staging Manual (7th edition, 2010)

### Effect of marital status on overall survival (OS)

**Figure 1:**
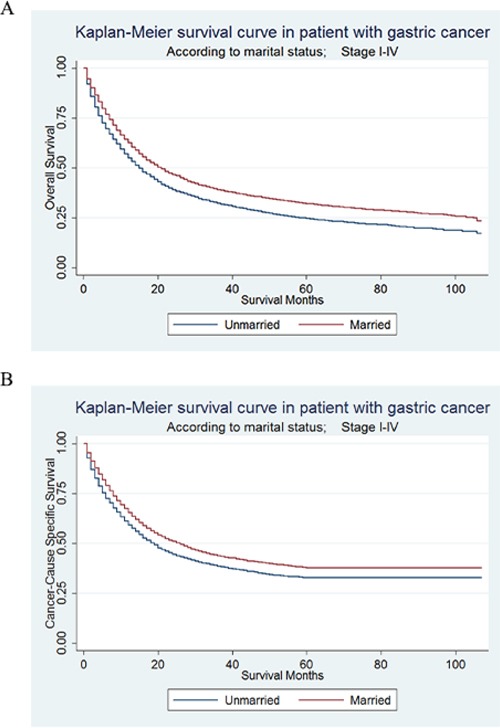
Kaplan-Meier Survival curves: The overall and cancer-caused specific survival of patients with gastric cancer according to marital status, unmarried versus married patients **A**. OS: χ^2^= 121.4, P<0.001; **B**. CSS: χ^2^= 66.42, P<0.001.

Kaplan–Meier curves were used to evaluate OS of GC patients (Figure 1A). The 5-year OS rate was 24.61% in the unmarried group and 32.09% in the married group. Married patients had better OS than the unmarried, which were significant according to the univariate log-rank test (P < 0.001) (Supplementary Table S1). In univariate analysis, other significant factors associated with OS included primary site, race, age, grade, histotype, TNM stage, surgery type and selection of radiotherapy. When these significant variables in univariate analysis were included and adjusted in the multivariate analysis with Cox regression, marital status was validated as an independent prognostic factor and marriage was found to be a protective factor from GC (HR = 0.88, 95% CI 0.85-0.92, P < 0.001).

Additionally, primary site, race, age, grade, histotype, TNM stage, surgery type and selection of radiotherapy were also independent prognostic factors for OS in the multivariate analysis.

### Effect of marital status on cancer-caused special survival (CSS)

CSS of GC patients were also calculated by Kaplan–Meier curve (Figure 1B). The 5-year CSS rate of the unmarried group was 32.79%, while the 5-year CSS rate of the married group was 37.74%. In univariate log-rank test, married patients had significantly better CSS than the unmarried (P < 0.001) (Table 2). Besides, primary site, sex, race, age, grade, histotype, TNM stage, surgery type and selection of radiotherapy were all proved to be significantly associated with CSS in univariate analysis. When all variables mentioned above were adjusted in the multivariate analysis with Cox regression, marital status was defined as independent prognostic factors and marriage was found to be a protective factor from GC (HR = 0.89, 95% CI 0.85-0.93, P < 0.001).

**Table 2:** Univariate and multivariate survival analysis for gastric cancer-caused special survival (CSS) predictors. SEER 2004-2012 (n=16910)

Variable	5-year CSS	Univariate analysis		Multivariate analysis		
		Log rank χ2	P value	HR	95%CI	P value
**Marital Status**		66.42	<0.001			
Unmarried	32.79%			Reference		
Married	37.74%			0.89	0.85-0.93	<0.001
**Site**		529.04	<0.001			
Fundus of stomach	27.67%			Reference		
Body of stomach	35.58%			0.93	0.83-1.04	0.197
Gastric antrum	41.64%			0.90	0.81-1	0.042
Pylorus	36.63%			1.01	0.88-1.15	0.912
Lesser curvature of stomach, NOS	45.52%			0.78	0.7-0.88	<0.001
Greater curvature of stomach, NOS	37.27%			0.98	0.86-1.12	0.797
Overlapping lesion of stomach	21.51%			1.06	0.95-1.18	0.331
Stomach, NOS	25.68%			1.09	0.98-1.21	0.106
**Sex**		4.1	0.043			
Male	36.70%			Reference	-	
Female	34.57%			0.95	0.91-0.99	0.022
**Race**		209.77	<0.001			
white	32.85%			Reference		
black	32.29%			1.07	1.01-1.14	0.021
American Indian or Alaska Native	28.98%			1.18	0.95-1.45	0.128
Asian or Pacific Islander	46.25%			0.81	0.76-0.86	<0.001
Unknown	65.02%			0.42	0.22-0.78	0.006
**Age**		97.94	<0.001			
18-27	15.66%			Reference		
28-37	30.28%			0.75	0.55-1.02	0.066
38-57	35.36%			0.81	0.61-1.08	0.147
58-69	38.75%			0.91	0.69-1.21	0.521
70-84	36.03%			1.21	0.91-1.6	0.195
85+	29.03%			1.80	1.35-2.41	<0.001
**Grade**		515.51	<0.001			
Grade I (well differentiated)	68.55%			Reference		
Grade II (moderately differentiated)	47.22%			1.34	1.13-1.59	0.001
Grade III ( poorly differentiated)	31.62%			1.75	1.47-2.07	<0.001
Grade IV (undifferentiated)	26.53%			2.10	1.68-2.61	<0.001
cell type not determined	26.04%			1.56	1.31-1.87	<0.001
**Histotype**		332.55	<0.001			
Adenocarcinoma, NOS	35.43%			Reference		
Adenocarcinoma, intestinal type	51.76%			0.83	0.77-0.9	<0.001
Carcinoma, diffuse type	31.43%			1.07	0.98-1.18	0.139
Tubular adenocarcinoma	44.28%			1.11	0.87-1.42	0.414
Papillary adenocarcinoma, NOS	48.20%			0.80	0.47-1.39	0.432
Mucinous adenocarcinoma	37.49%			1.00	0.85-1.18	0.994
Signet ring cell carcinoma	28.16%			1.13	1.07-1.19	<0.001
**TNM Stage^c^**		4727.01	<0.001			
Stage I	66.56%			Reference		
Stage II	46.47%			1.99	1.84-2.16	<0.001
Stage III	24.44%			3.71	3.44-4.02	<0.001
Stage IV	4.36%			5.03	4.67-5.43	<0.001
**Surgery**		4047.32	<0.001			
No surgery	6.16%			Reference		
Non-Total or Non-near-total gastrectomy	49.12%			0.34	0.32-0.36	<0.001
Total or near total gastrectomy	37.03%			0.41	0.38-0.44	<0.001
**Radiation**		172.16	<0.001			
No radiotherapy	34.33%			Reference		
Radiotherapy	40.23%			0.76	0.72-0.8	<0.001
Radiotherapy unknown	34.68%			0.87	0.73-1.05	0.138

Abbreviation: NOS= no other specific; SEER=Surveillance, Epidemiology and End Results.

In addition to marital status, primary site, sex, race, age, grade, histotype, TNM stage, surgery type and selection of radiotherapy were also independent prognostic factors for CSS in the multivariate Cox regression analysis.

### Survival analysis in matched group

In order to reduce potential selection bias, we used the propensity score matching method to carry out a 1:1 matched case-control analysis. Each unmarried patient was matched to one married patient, according to histological grade, primary site, and TNM stage. The detailed information was shown in Supplementary Table S2. We enrolled 14056 patients and 7028 for each group. After matching, there was no significant difference in baseline characteristics including primary site (P=0.054), TNM stage (P=0.057) and histological grade (P=0.712). Kaplan–Meier curves were shown in Supplementary Figure S2 and married patients still had a better CSS than unmarried patients (5-year CSS: 36.54% versus 32.79%) by univariate log-rank analysis (P<0.001), as well as multivariate cox analysis (HR=0.90, 95%CI=0.85-0.94, P<0.001) (Supplementary Table S3). This result proved that our analysis was credible, which meant that the selection bias was not a source of error.

### Subgroup analyses

**Figure 2:**
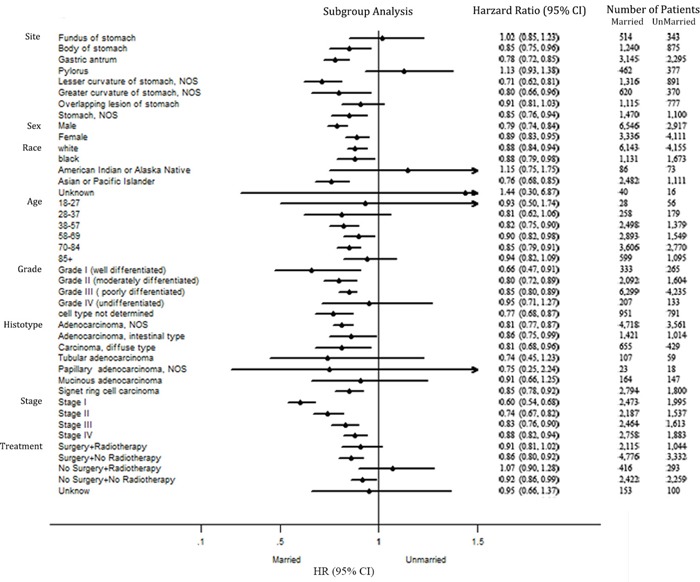
Forest plot summarizing hazard ratios for married versus unmarried patients in subgroup analyses The X-axis displays the hazard ratio and 95% CI of each subgroup, ticks are arranged at 0.1, 0.5, 1.0 and 1.5.

A forest plot of the HRs summarized exploratory subgroup analyses for CSS in Figure 2. We assigned the types of treatments into groups by combination surgery and radiotherapy as both surgery and radiotherapy, surgery without radiotherapy, no surgery but radiotherapy, neither surgery nor radiotherapy and unknown treatment. The result of subgroup analyses indicated that marriage was no longer protective factors in some subgroups. We suggested that primary site and race might be important confounders for effect of marital status in GC prognosis.

### Effect of marital status on stage at diagnosis

We hypothesized that marital status might affect survival of GC patients in the aspects of stage at diagnosis. If it was true, compared with married group, unmarried patients should be more likely to be diagnosed at later stage. Table 3 presented the patients’ characteristics by stage at diagnosis. Early and late stage corresponded to stage I-II and stage III-IV, respectively. The relationship between marital status and stage at diagnosis was analyzed by univariate and multivariate logistic regression models. In univariate analysis, in addition to marital status (P < 0.001), we also found that primary site, race, age, grade, and histotype were all associated with stage at diagnosis. After adjustment of these confounding variables in multivariable logistic test, married patients were not significantly more likely to be diagnosed at early stage (P=0.159) compared with unmarried patients.

**Table 3:** Characteristics of patients by stage at diagnosis with corresponding univariate and multivariate analysis. SEER 2004-2012 (n=16910)^a^

Characteristics	Early stage^b^	Late stage^c^	Univariate analysis	Multivariate analysis		
	8192 (100)	8718(100)	P value^d^	OR	95%CI	P value^e^
**Marital Status**			<0.001	Reference		
Unmarried	3532 (43.12)	3496(40.1)				
Married	4660 (56.88)	5222(59.9)		1.05	0.98-1.12	0.159
**Site**			<0.001			
Fundus of stomach	403 (4.92)	454(5.21)		Reference		
Body of stomach	1061 (12.95)	1054(12.09)		0.87	0.74-1.02	0.094
Gastric antrum	2878 (35.13)	2562(29.39)		0.83	0.72-0.97	0.018
Pylorus	432 (5.27)	407(4.67)		0.86	0.71-1.05	0.137
Lesser curvature of stomach, NOS	1197 (14.61)	1010(11.59)		0.75	0.64-0.89	0.001
Greater curvature of stomach, NOS	501 (6.12)	489(5.61)		0.84	0.7-1.02	0.077
Overlapping lesion of stomach	629 (7.68)	1263(14.49)		1.64	1.38-1.95	<0.001
Stomach, NOS	1091 (13.32)	1479(16.96)		1.13	0.97-1.33	0.126
**Sex**			0.0864			
Male	4529 (55.29)	4934(56.6)				
Female	3663 (44.71)	3784(43.4)				
**Race**			<0.001			
white	4839 (59.07)	5459(62.62)		Reference		
black	1367 (16.69)	1437(16.48)		0.94	0.86-1.03	0.168
American Indian or Alaska Native	74 (0.9)	85(0.97)		0.99	0.71-1.38	0.961
Asian or Pacific Islander	1879 (22.94)	1714(19.66)		0.85	0.78-0.92	<0.001
Unknown	33 (0.4)	23(0.26)		0.56	0.32-0.97	0.039
**Age**			<0.001			
18-27	25 (0.31)	59(0.68)		Reference		
28-37	129 (1.57)	308(3.53)		1.01	0.6-1.7	0.961
38-57	1450 (17.7)	2427(27.84)		0.78	0.49-1.26	0.318
58-69	1999 (24.4)	2443(28.02)		0.63	0.39-1.01	0.057
70-84	3425 (41.81)	2951(33.85)		0.47	0.29-0.76	0.002
85+	1164 (14.21)	530(6.08)		0.25	0.15-0.4	<0.001
**Grade**			<0.001			
Grade I (well differentiated)	499 (6.09)	99(1.14)		Reference		
Grade II (moderately differentiated)	2292 (27.98)	1404(16.1)		3.11	2.47-3.9	<0.001
Grade III ( poorly differentiated)	4480 (54.69)	6054(69.44)		5.50	4.4-6.88	<0.001
Grade IV (undifferentiated)	138 (1.68)	202(2.32)		5.61	4.1-7.67	<0.001
cell type not determined	783 (9.56)	959(11)		4.77	3.75-6.07	<0.001
**Histotype**			<0.001			
Adenocarcinoma, NOS	4131 (50.43)	4148(47.58)		Reference		
Adenocarcinoma, intestinal type	1510 (18.43)	925(10.61)		0.75	0.68-0.82	<0.001
Carcinoma, diffuse type	421 (5.14)	663(7.6)		1.10	0.96-1.26	0.186
Tubular adenocarcinoma	105 (1.28)	61(0.7)		0.80	0.57-1.11	0.175
Papillary adenocarcinoma, NOS	31 (0.38)	10(0.11)		0.44	0.21-0.93	0.031
Mucinous adenocarcinoma	144 (1.76)	167(1.92)		1.26	1-1.6	0.054
Signet ring cell carcinoma	1850 (22.58)	2744(31.48)		0.99	0.91-1.07	0.827

Abbreviation: NOS= no other specific; SEER=Surveillance, Epidemiology and End Results.

^a^Data are presented as No.(percentage) of patients.

^b^Early stage is stage I and stage II according to the criteria of AJCC Cancer Staging Manual (7th edition, 2010).

^c^Late stage is stage III and stage IV according to the criteria of AJCC Cancer Staging Manual (7th edition, 2010).

^d^P values are from univariate logistic tests.

^e^P values are from a multivariable logistic test.

### Subgroup analysis for evaluating the effect of marital status on OS and CSS according to stage at diagnosis

Another hypothesis is that post-diagnosis factors including choice of treatment and social support play a role in the effect of marital status on survival. If this hypothesis was wrong, namely that marital status only affected survival of GC patients in aspects of stage at diagnosis, marital status would not affect survival after the tumor was diagnosed. Further explorations were made by us to identify the difference between married and unmarried groups in each stage subgroup.

**Figure 3:**
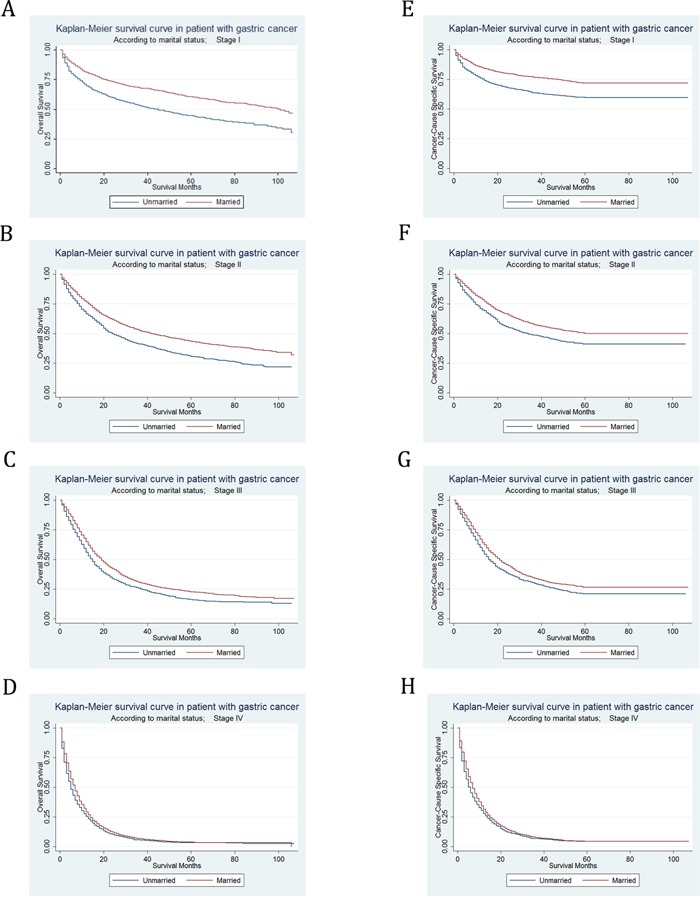
Kaplan-Meier Survival curves: The overall and cancer-caused specific survival of patients with gastric cancer according to marital status by TNM stage at diagnosis **A**. OS at stage I: χ^2^= 105.13, P<0.001; **B**. OS at stage II: χ^2^= 60.38, P<0.001; **C**. OS at stage III: χ^2^= 31.99, P<0.001; **D**. OS at stage IV: χ^2^= 16.34, P<0.001; **E**. CSS at stage I: χ^2^= 72.52, P<0.001; **F**. CSS at stage II: χ^2^= 33.32, P<0.001; **G**. CSS at stage III: χ^2^= 20.51, P<0.001; **H**. CSS at stage IV: χ^2^= 14.6, P<0.001.

In stage I, the 5-year OS rate of the unmarried group was 44.59%, while the 5-year OS rate of the married group was 60.60%; the 5-year CSS rate of the unmarried group was 59.61%, while the 5-year CSS rate of the married group was 71.86%. In stage II, the 5-year OS rate of the unmarried group was 30.78%, while the 5-year OS rate of the married group was 43.39%; the 5-year CSS rate of the unmarried group was 41.20%, while the 5-year CSS rate of the married group was 49.95%. In stage III, the 5-year OS rate of the unmarried group was 16.03%, while the 5-year OS rate of the married group was 22.70%; the 5-year CSS rate of the unmarried group was 21.01%, while the 5-year CSS rate of the married group was 26.51%. In stage IV, the 5-year OS rate of the unmarried group was 3.33%, while the 5-year OS rate of the married group was 3.50%; the 5-year CSS rate of the unmarried group was 4.31%, while the 5-year CSS rate of the married group was 4.44%. We found that marital status was significantly associated with either OS (Supplementary Table S4) or CSS (Table 4) in each tumor stage by univariate log-rank analysis. Kaplan–Meier curves were shown in Figure 3.

**Table 4:** Univariate and multivariate survival analysis for marital status on gastric cancer-caused special survival (CSS) by stage at diagnosis. SEER 2004-2012 (n=16910)

TNM stage	Univariate analysis			Multivariate analysis		
	5-year CSS	Log rank χ2	P value	HR	95%CI	P value
**Stage I(n=4468)**						
**Marital status**		72.52	<0.001			
Unmarried	59.61%			Reference		
Married	71.86%			0.95	0.84-108	0.407
**Stage II(n=3, 724)**						
**Marital status**		33.32	<0.001			
Unmarried	41.20%			Reference		
Married	49.95%			0.88	0.79-0.98	0.016
**Stage III(n=4077)**						
**Marital status**		20.51	<0.001			
Unmarried	21.01%			Reference		
Married	26.51%			0.83	0.73-0.93	0.002
**Stage IV(n=4641 )**						
**Marital status**		14.6	<0.001			
Unmarried	4.31%			Reference		
Married	4.44%			0.92	0.86-0.99	0.024

Abbreviation: SEER=Surveillance, Epidemiology and End Results.

However, after adjusting sex, race, age, grade, histotype, surgery type and selection of radiotherapy in multivariate analysis, the marital status was no longer an independent prognostic factor in stage I subgroup for OS (HR=0.93, 95% CI 0.84-1.03, P = 0.139) or CSS (HR=0.95, 95% CI 0.84-1.08, P = 0.407). In spite of this, for both OS and CSS, marital status was still defined as an independent prognostic factor and marriage was found to be a protective factor from GC in stage II (OS, HR=0.85, 95% CI 0.78-0.94, P = 0.001; CSS, HR=0.88, 95% CI 0.79-0.98, P = 0.016), stage III (OS, HR=0.88, 95% CI 0.82-0.96, P = 0.003; CSS, HR=0.83, 95% CI 0.73-0.93, P = 0.002), and stage IV (OS, HR=0.92, 95% CI 0.86-0.99, P = 0.018; CSS, HR=0.92, 95% CI 0.86-0.99, P = 0.024) in multivariate Cox regression analysis.

### Effect of marital status on selection of treatment

To prove that marital status could affect survival by treatment, we then did research on the effect of marital status on selection of treatment. Supplementary Table S5 & Supplementary Table S6 showed the patients’ characteristics by selection of surgery and radiotherapy. The relationship between marital status and treatment at diagnosis was analyzed by univariate and multivariate multinomial logistic regression models. In univariate analysis, marital status, age, primary site, race, age, grade, and histotype were associated with selection of surgery, while marital status age, sex, primary site, race, age, grade, and histotype were associated with selection of radiotherapy. After adjustment of confounding factors mentioned above in multivariable logistic test, we found that married patients were more likely than unmarried to undergo non-total gastrectomy (RRR=1.34, 95% CI 1.23-1.45, P < 0.001) or total gastrectomy (RRR=1.38, 95% CI 1.24-1.53, P < 0.001) rather than no surgery (Table 5), but yet no significant difference was recognized between non-total gastrectomy and total gastrectomy by marital status (P=0.55) (Supplementary Table S7). In the meanwhile, married patients were more likely than unmarried to undergo radiotherapy (RRR=1.29, 95% CI 1.19-1.39, P < 0.001) rather than no radiotherapy (Table 6).

**Table 5:** Multinomia multivariate analysis of surgery by marital status, compared to patients with no surgery. SEER 2004-2012 (n=16910)

Surgery	Multivariate analysis		
	RRR	[95%CI]	P value
**No surgery**	(base outcome)		
**Marital Status**			
Unmarried			
Married			
**Non-Total or Non-near-total gastrectomy**			
**Marital Status**			
Unmarried	Reference		
Married	1.34	1.23-1.45	<0.001
**Total or near total gastrectomy**			
**Marital Status**			
Unmarried	Reference		
Married	1.38	1.24-1.53	<0.001

Abbreviation: SEER=Surveillance, Epidemiology and End Results.

**Table 6:** Multinomia multivariate analysis of radiotherapy by marital status, compared to patients with no radiotherapy. SEER 2004-2012 (n=16910)

Radiotherapy	Multivariate analysis		
	RRR	[95%CI]	P value
**No radiotherapy**	(base outcome)		
**Marital Status**			
Unmarried			
Married			
**Radiotherapy**			
**Marital Status**			
Unmarried	Reference		
Married	1.29	1.19-1.39	<0.001
**Radiotherapy unknown**			
**Marital Status**			
Unmarried	Reference		
Married	1.06	0.81-1.39	0.667

Abbreviation: SEER=Surveillance, Epidemiology and End Results.

### Subgroup analysis for evaluating the effect of marital status on OS and CSS according to treatment

OS and CSS were respectively compared between married and unmarried patients in each subgroup of treatment, in order to certify that other post-diagnosis factors such as social support also influenced the effect of marital status on survival. If it was true, marital status would still effect survival of GC patients after treatment.

**Figure 4:**
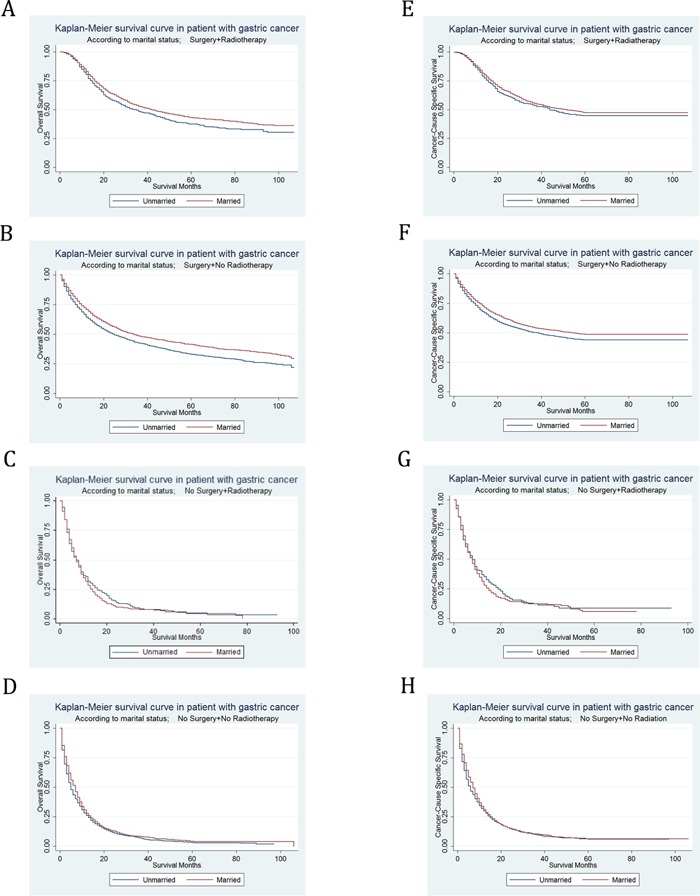
Kaplan-Meier Survival curves: The overall and cancer-caused specific survival of patients with gastric cancer according to marital status by treatments **A**. OS, surgery and radiotherapy: χ^2^= 8.81, P=0.003; **B**. OS, surgery without radiotherapy: χ^2^= 45.31, P<0.001; **C**. OS, no surgery but radiotherapy: χ^2^= 0.74, P=0.3897; **D**. OS, no surgery or radiotherapy: χ^2^= 10.59, P=0.0011; **E**. CSS, surgery and radiotherapy: χ^2^= 2.57, P=0.1086; **F**. CSS, surgery without radiotherapy: χ^2^= 18.74, P<0.001; **G**. CSS, no surgery but radiotherapy: χ^2^= 0.69, P=0.4069; **H**. CSS, no surgery or radiotherapy: χ^2^= 5.58, P=0.0182.

With Kaplan–Meier curves, we estimated OS and CSS by marital status in subgroups of treatment (Figure 4). In patient who underwent both surgery and radiotherapy, the 5-year OS rate of the unmarried group was 37.59%, while the 5-year OS rate of the married group was 43.12%; the 5-year CSS rate of the unmarried group was 44.65%, while the 5-year CSS rate of the married group was 47.38%. In patients who underwent surgery without radiotherapy, the 5-year OS rate of the unmarried group was 33.06%, while the 5-year OS rate of the married group was 41.24%; the 5-year CSS rate of the unmarried group was 44%, while the 5-year CSS rate of the married group was 48.73%. In patient who underwent no surgery but radiotherapy, the 5-year OS rate of the unmarried group was 4.7%, while the 5-year OS rate of the married group was 4.53%; the 5-year CSS rate of the unmarried group was 8.38%, while the 5-year CSS rate of the married group was 5.73%. In patient who underwent neither surgery nor radiotherapy, the 5-year OS rate of the unmarried group was 3.11%, while the 5-year OS rate of the married group was 4.08%; the 5-year CSS rate of the unmarried group was 5.82%, while the 5-year CSS rate of the married group was 6.17%. We found that marital status was significantly associated with OS in patient who underwent both surgery and radiotherapy, surgery without radiotherapy and neither surgery nor radiotherapy (P=0.0011) by univariate log-rank analysis (Supplementary Table S8). Meanwhile, in univariate log-rank analysis, marital status was significantly associated with CSS in patient who underwent surgery without radiotherapy (P < 0.001), and neither surgery nor radiotherapy (P=0.0182) (Table 7).

**Table 7:** Univariate and multivariate survival analysis for marital status on gastric cancer-caused special survival (CSS) by treatment. SEER 2004-2012 (n=16910)

Treatment	Univariate analysis			Multivariate analysis		
	5-year CSS	Log rank χ2	P value	HR	95%CI	P value
**Surgery and radiotherapy (n=3159)**						
**Marital Status**		2.57	0.109			
Unmarried	44.65%					
Married	47.38%					
**Surgery without radiotherapy (n=8108)**						
**Marital Status**		18.74	<0.001			
Unmarried	44.00%			Reference		
Married	48.73%			0.89	0.83-0.96	0.003
**No surgery but radiotherapy (n=709)**						
**Marital Status**		0.69	0.407			
Unmarried	8.38%					
Married	5.73%					
**No surgery or radiotherapy (n=4681)**						
**Marital Status**		5.58	0.018			
Unmarried	5.82%			Reference		
Married	6.17%			0.88	0.81-0.95	0.001
**Unknown (n=253)**						
**Marital Status**		0.09	0.762			
Unmarried	38.29%					
Married	33.22%					

Abbreviation: NOS= no other specific; SEER=Surveillance, Epidemiology and End Results.

Then we used multivariate Cox regression analysis to adjust confounding factors including sex, race, age, grade, histotype, TNM stage. It was found that the marital status was no longer an independent prognostic factor for OS in patients who underwent both surgery and radiotherapy (HR=0.92, 95% CI 0.83-1.03, P = 0.131). Yet for all that, for both OS and CSS, marital status was still certificated as independent prognostic factors and marital status was found to be a protective factor from GC in patient who undergo surgery without radiotherapy (OS, HR=0.86, 95% CI 0.81-0.92, P < 0.001; CSS, HR=0.89, 95% CI 0.8-0.96, P = 0.003), and neither surgery nor radiotherapy (OS, HR=0.87, 95% CI 0.81-0.94, P < 0.001; CSS, HR=0.88, 95% CI 0.81-0.95, P = 0.001) in multivariate analysis.

## DISCUSSION

A great number of researches focused on the influence of marital status on urogenital neoplasms. Osborne et al. reported that older married women had a lower risk of mortality after a diagnosis of breast cancer.[11] Among women with cervical cancer, marital status was reported to interact with tumor stage and radiotherapy to effect survival, instead of an independent prognostic factor.[17] Krongrad reported that married patients with prostate cancer had better survival than those who were single, divorced, separated or widowed.[18] As a non-urogenital system cancer, the influence of marital status on GC was reported in few studies, and no previous study reported the effect of marital status on CSS of GC patients or its underlying mechanism based on a large population. Using a population-based cohort of adult patients with GC, we indicated that marriage is an independent protective prognostic factor for OS and CSS, which was controversial in previous researches.[9, 13, 14] Besides, we studied the influence of marital status on stage at diagnosis, treatment, and survival in each subgroup of stage or treatment. As far as we know, it is the first study to shed light on the deep mechanisms how marital status affects OS and CSS of GC patients.

The first hypothesis to explain why married GC patients had better survival was that married patients preferred earlier screening and diagnosis. However, married patients didn't present higher probability to be diagnosed at early stage. Screening of the high-risk population in US may contribute to this result.[19] This finding suggests that delayed diagnosis is not a reason for poor prognosis in unmarried patients.

In our analysis, married patients demonstrated both OS and CSS advantages in each subgroup of stage II-IV, but not in stage I. It could be explained that unlike the patient diagnosed at late stage, these patients do not require adjuvant therapy or social support in early stage. The result indicated that in stage II-IV, post-diagnosis factors could be possible mechanisms of effect of marital status on survival, such as treatment and social support.

Secondly, we hypothesized that poor survival outcomes in unmarried patients were attributed to undertreatment. Compared with unmarried patients, married patients were more likely to undergo surgery rather than no surgery, but no significant difference was defined between different surgery methods (non-total gastrectomy and total gastrectomy) by marital status. In the meanwhile, married patients were more likely to undergo radiotherapy rather than no radiotherapy. The difference of treatment by marital status showed that unmarried patients were less likely to receive treatments, compared with married patients. Obviously, this result demonstrates that undertreatment contributes to poor prognosis in unmarried group.

Finally, to explore if there was any post-diagnosis factor beyond treatment like social support acting as a possible mechanism of effect of marital status on survival, we regrouped the selection of treatments and compared OS and CSS of different marital status in each subgroup. After adjustment for possible confounders, we observed some interesting findings. First, marital status was an independent prognostic factor in patients who underwent surgery without radiotherapy, but not in patients underwent both surgery and radiotherapy. It could be explained that in patients treated with surgery, simultaneous reception of radiotherapy always suggests better financial situation, and stronger will of active treatments.[20, 21] In view of these factors, the results of surgeries on these patients are usually satisfactory, so they do not require extra social support. Moreover, marriage is not a real reflection of economic status or much social support in this group owing to above factors. In another group without radiotherapy, we could treat patients as two kinds. On whom have good better financial situation but diffused tumors not suitable for radiotherapy, the surgeries have only limited effect, even if these patients are willing to receive active treatments. Hence, they require more social support such as health care and psychosocial support, which are associated with marriage.[22–25] On the other hand, for patients with limited tumor, marital status is relevant to much social support like economic status, health care, psychosocial support for them. It was reported that married ones had healthier lifestyle than the unmarried.[25] These explained that marital status was an independent prognostic factor in patients who underwent surgery without radiotherapy.

The second interesting finding was that in patients who didn't receive any treatment (neither surgery nor radiotherapy), marital status was an independent protective factor for both OS and CSS, even after adjustment for confounders including primary site, sex, race, age, grade, histotype, and TNM stage at diagnosis. We supposed that these patients are not suitable for any treatment or can't afford it. On the condition of undertreatment, more additional social support is required.

The benefits of marriage to the survival of GC patients after adjusting diagnosis and treatment in our study have a variety of potential underlying etiologies. Psychologically, married population was reported to possess lower level of distress and depression after diagnosis, hence spouses could share the emotional burden and provide psychological support.[22–24] In the previous researches, decreased social support and psychological stress were found to activate specific signaling pathways in immunosuppression and lead to tumor growth and progression.[26–30] Some studies reported that hypothalamic-pituitary-adrenal (HPA) function was activated to suppress T-cell mediated immune responses in depressed population.[31] Low expression of IFN-c, CCL27/CTACK, and CD3ε gene, as well as low infiltration of CD4+ and CD8+ T cells within and around tumors are related to stressed group.[26] Levy et al. reported that social support could act as a predictor of natural killer cell activity in patients with breast cancer.[32] Additionally, cortisol is secreted by the adrenal cortex in response to stress,[33, 34] while social support and reduced stress are associated with the level of cortisol.[35–37] Arranz et al. reported that peripheral CRF contributed to proliferation and metastasis of tumors, by means of altering the expression of SMAD2, β-catenin, and cytoskeletal genes.[38] As proangiogenic factors that play a considerable role in neovascularization and development of GC[39–41], lower levels of VEGF and IL-6 are shown to have links with a higher level of social support.[42–44] It had also been proposed that norepinephrine (NE), a stress hormones, led to anoikis avoidance and metastasis mediated by ADRB2.[45]

Socioeconomically, marriage reflects better economic status, which was reported to increase the survival of cancers.[46, 47] Higher nursing and medical levels are also consequences upon better economic status. Besides, marriage was reported to give rise to increasing adherence to medical regimens due to social support.[48, 49] At the same time, married patients can receive extra health care from spouses. Reduced cancer-related lifestyles and more healthy lifestyles are shown among married population.[25, 50] Many researches indicated that wholesome behaviors were favorable prognostic factors for GC, including frequent intaking of raw vegetable and fruit, no smoking habit, and daily physical activities.[51–53]

The marital status was categorized into married and unmarried groups in our study. The latter consisted of single, divorced or separated, and widowed groups, among which there was no difference of CSS in univariate log-rank test (P= 0.1626), hence we put them in the same class as unmarried group. Meanwhile, grouping as a binary variable could underline the effect of factors that were different between married and unmarried population, such as support of family and financial situation.[54]

Inevitably, our study had several potential limitations. Firstly, the information of chemotherapy, other types of therapy, and quality of treatments was not accessible in the SEER database. We couldn't adjust these factors in the effect of marriage on survival. Secondly, marital status might change after diagnosis and influence survival, but SEER database only provided the marital status at diagnosis. Thirdly, the information of income, education, marital satisfaction, and insurance status was lacking, which could all affect survival of GC patient and be confound factors in our analysis.[14, 55–59] Fourthly, we couldn't confirm the specific mechanism by which marriage enhanced survival in GC apart from treatment. We mentioned that the etiologies of marital status effect on better survival included psychological support, extra health care, increasing adherence to medical regimens and healthy lifestyles. Nonetheless, we couldn't analyze the relationship between these factors and survival due to lack of statistics. Fifthly, cohabitation wasn't recorded in SEER and only patients with legal marriage were classified in married group. The social support from cohabitating partner wasn't taken into account in this study. Sixthly, our conclusions can only be applied in US, due to socioeconomic and medical disparities among countries. Finally, we can't ascertain the reason for fewer treatments in unmarried people, because both doctors’ recommends and patients’ refusals are likely to contribute to the result.

In spite of these potential limitations, our study shed new light on the mechanisms how marital status affects both OS and CSS in GC patients, with data from the large population-based SEER database. We support the conclusion that marital status is an independent good prognostic factor for survival of adult patients with GC, and the same result was confirmed in stage subgroup II-IV. Moreover, we found heterogeneous effect of marriage on GC patients’ survival in subgroups of stage and treatment. We didn't find the association between marriage and earlier stage at diagnosis, namely delayed diagnosis didn't account for poor survival in unmarried patients. More importantly, our result illuminated that undertreatment was an important mechanism of effect of marital status on survival, since unmarried patients were less likely to undergo surgery or radiotherapy. Married patients had better survival even after controlling for stage at diagnosis and treatment, indicating social support was also a possible mechanism. As we mentioned, marriage was known as the most important social support. Lack of psychological and socioeconomic factors provided by marriage may explain the poor survival outcomes in unmarried patients without receiving radiotherapy. The direct effects of these factors need to be clarified in further researches. Identifying the impact of marital status on survival of GC patients and the underlying mechanisms, we could carry out corresponding strategies to prevent disease progression and increase survival. More psychological care and social support are needed for unmarried patients with GC as long ago as they made decision on treatments, especially who were diagnosed at late stage and underwent no treatment.

## MATERIALS AND METHODS

### Data source

We used Surveillance, Epidemiology, and End Results (SEER) database released in April 2015 as data source, which includes data from 18 population-based registries from 1973 to 2012 and covers approximately 30% of the population in the US. The SEER Program registries routinely collect data on patient demographics, primary tumor site, tumor morphology and stage at diagnosis, first course of treatment, and follow-up for vital status. The mortality data reported by SEER are provided and updated annually by the National Center for Health Statistics.[60]

### Inclusion criteria

To identify appropriate patients for this study, we used inclusion criteria as follows: Patients were aged 18 or older at diagnosis with primary gastric cancer (International Classification of Diseases for Oncology, Third Edition [ICD-O-3], codes C16.1-C16.6, C16.8-C16.9). Tumors located at the cardia or esophagogastric junction site (ICD-O-3 site code C16.0) were excluded due to different incidence and etiology.[61] Histological types included adenocarcinoma (ICD-O-3 8140/3, 8144/3, 8145/3), tubular adenocarcinoma (ICD-O-3 8211/3), papillary adenocarcinoma (ICD-O-3 8260/3), mucinous adenocarcinoma (ICD-O-3 8480/3) and signet ring cell carcinoma (8490/3). All patients had histological confirmation of diagnosis besides autopsy and death certificate, and were actively followed up. Patients were excluded if they had unknown marital status, undefined TNM stage, unknown cause of death or unknown survival months. Patients who were diagnosed before 2004 and after 2012 were excluded, because 6^th^ or 7^th^ edition of the American Joint Committee on Cancer (AJCC) Cancer Staging Manual were not recorded before 2004, and the SEER database was only updated up to December 31, 2012. Finally, 16910 patients were included into our study.

### Study variables

Variables extracted from the SEER database included marital status, sex, race, age at diagnosis, histotype, histological grade, primary site, TNM stage, cause of death, selection of surgery, and selection of radiotherapy.

Marital status at diagnosis was categorized as a binary variable into married and unmarried (including single, divorced or separated, and widowed groups). Race was classified as white, black, American Indian or Alaska Native, Asian or Pacific Islander, and others. We divided age at diagnosis into groups: 18 to 27 year, 28 to 37 year, 38 to 57 year, 58 to 69 year, 70 to 84 year, 85 year or over. Data of histotype, histology grade and primary site were all coded according to ICD-O-3. Primary site was categorized as fundus of stomach; body of stomach; gastric antrum; pylorus; lesser curvature of stomach, no otherwise specified (NOS); greater curvature of stomach, NOS; overlapping lesion of stomach; stomach, NOS. Stage at diagnosis was restaged according to the criteria of AJCC Cancer Staging Manual (7th edition, 2010).[62, 63] We separated the selection of surgery into following groups: no surgery, non-Total or non-near-total gastrectomy, and total or near total gastrectomy. Similarly, the selection of radiotherapy was categorized as no radiotherapy, radiotherapy, and radiotherapy unknown. In subgroup analysis, we assigned treatments into groups by combination surgery and radiotherapy as both surgery and radiotherapy, surgery without radiotherapy, no surgery but radiotherapy, neither surgery nor radiotherapy and unknown treatment.

### Outcome measurement

Outcomes of interest included overall survival (OS) and cancer-caused special survival (CSS). OS was calculated from the date of diagnosis to the date of death. Deaths of any cause were treated as events, while patients with longer survival times on the date of last contact were censored. CSS, as another outcome, was defined from the date of diagnosis to the date of death attributed to stomach cancer. Deaths attributed to GC were treated as events. Patients who died from other causes or were still alive at the time of the last follow-up were treated as censored observations. We took December 31, 2012 as the study cut-off date, because the seer database released in April 2015 didn't provide data after 2012.

### Statistical analyses

Clinicopathological baseline characteristics were compared with Pearson chi-square test for categorical data, and Wilcoxon-Mann-Whitney test for ranked data. The OS and CSS rate was calculated by Kaplan–Meier curve, and compared by Log-rank (Mantel-Cox) test. We presented a forest plot to summarize the hazard ratios of married versus unmarried patients in subgroups by univariate Cox regression analysis. Multivariate Cox proportional hazard models were built to determine risk factors of survival outcomes. The independent variables by stage at diagnosis and selection of treatment were analyzed by univariate and multivariate binomial or multinomial logistic regression models with adjustment for possible confounders.

In order to reduce selection bias, we used propensity score to carry out a matched case-control analysis. Psmatch2 is an extension packages in Stata and designed for the propensity score matching methods. We matched each unmarried patient to one married patient by Psmatch2, according to histological grade, primary site, and TNM stage.

All of data were analyzed by Stata statistical software, version 12.0 (StataCorp, College Station, TX). All P values were two-sided and statistical significance was set at P<0.05. All confidence intervals (CIs) were stated at the 95% confidence level.

## SUPPLEMENTARY MATERIALS FIGURES AND TABLES




