# Supplementary material for: The influence of marital status on the stage at diagnosis, treatment, and survival of adult patients with gastric cancer: a population-based study
Source: Oncotarget. 2016 Feb 15;8(14):22385–405. doi: 10.18632/oncotarget.7399 (PMC5410231; doi:10.18632/oncotarget.7399)
Supplement: Supplementary file 1 [file oncotarget-08-22385-s001.pdf]

SUPPLEMENTARY FIGURES AND TABLES

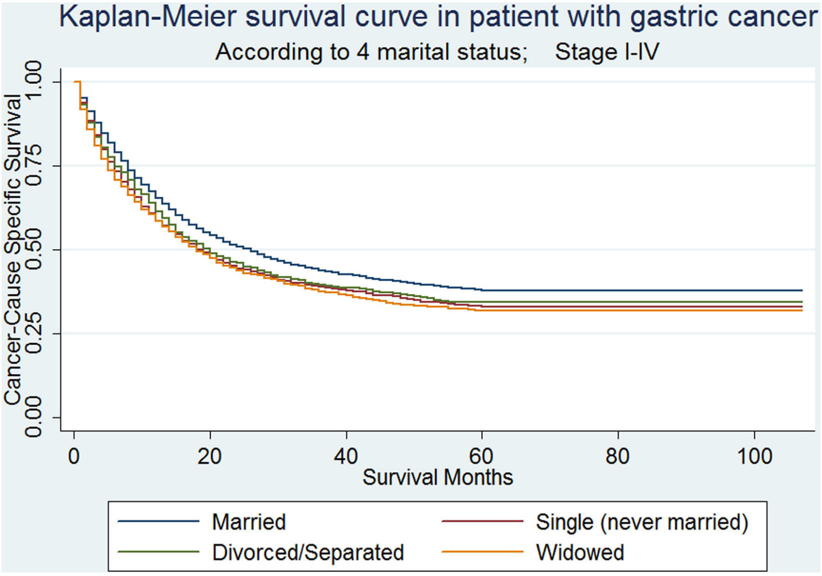

Supplementary Figure S1: Kaplan-Meier Survival curves: The cancer-specific survival of patients with gastric cancer by marital status, categorized into married, single (never married), divorced or separated and widowed groups. We compare the survival curves of single, divorced/separated, and widowed groups, among which there was no difference of CCS in univariate log-rank test (χ^2= 3.63, P=0.1626) (Figure 1), hence we put them in the same class as unmarried group.

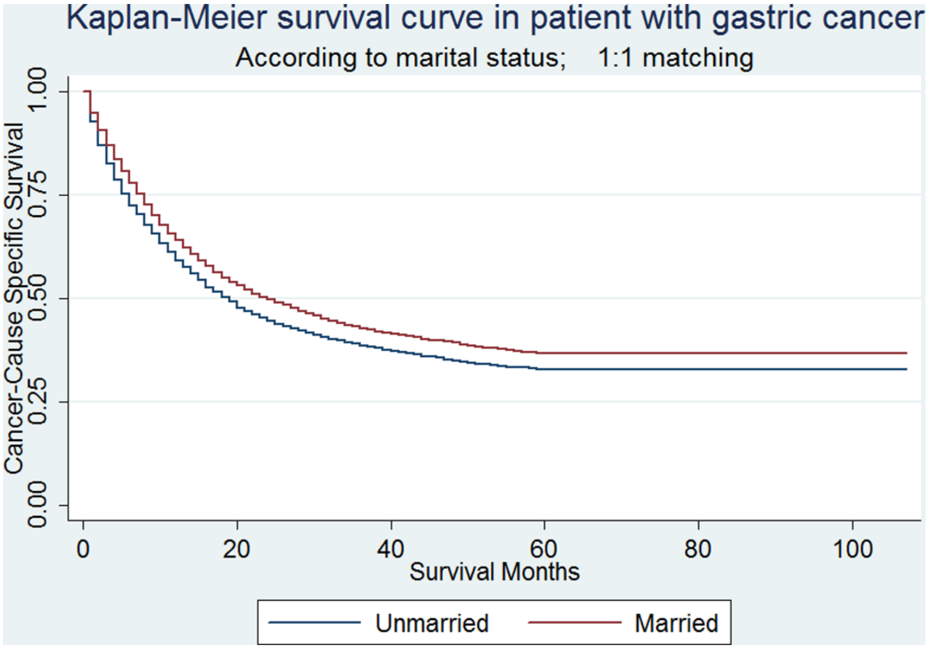

Supplementary Figure S2: Kaplan-Meier Survival curves: The cancer-specific survival of patients with gastric cancer by marital status in 1:1 matching, unmarried versus married patients. χ^2=35.35, P<0.001

Supplementary Table S1: Univariate and multivariate survival analysis for overall survival (OS) predictors. SEER 2004-2012 (n=16910)

See Supplementary File 1

Supplementary Table S2: Characteristics of patients by marital status in 1:1 matching, unmarried verus married. SEER 2004-2012 (n=14056)[a]

See Supplementary File 1

Supplementary Table S3: Univariate and multivariate survival analysis for gastric cancer-caused special survival (CSS) predictors in 1:1 matching. SEER 2004-2012 (n=14056)

See Supplementary File 1

Supplementary Table S4: Univariate and multivariate survival analysis for marital status on overall survival (OS) by stage at diagnosis. **SEER 2004-2012 (n=16910)**

TNM stage	Univariate analysis			Multivariate analysis		
	5-year OS	Log rank χ2	P value	HR	95%CI	P value
**StageI(n=4468)**						
**Marital status**		105.13	<0.001			
Unmarried	44.59%			Reference		
Married	60.60%			0.93	0.84-1.03	0.139
**StageII(n=3, 724)**						
**Marital status**		60.38	<0.001			
Unmarried	30.78%			Reference		
Married	43.39%			0.85	0.78-0.94	0.001
**StageIII(n=4077)**						
**Marital status**		31.99	<0.001			
Unmarried	16.03%			Reference		
Married	22.70%			0.88	0.82-0.96	0.003
**StageIV(n=4641)**						
**Marital status**		16.34	<0.001			
Unmarried	3.33%			Reference		
Married	3.50%			0.92	0.86-0.99	0.018

Abbreviation: NOS= no other specific; SEER=Surveillance, Epidemiology and End Results.

Supplementary Table S5: Characteristics of patients by surgery with corresponding multinomia univariate analysis. SEER 2004-2012 (n=16910)[a]

See Supplementary File 1

Supplementary Table S6: Characteristics of patients by radiotherapy with corresponding multinomia univariate analysis. SEER 2004-2012 (n=16910)[a]

See Supplementary File 1

Supplementary Table S7: Multinomia multivariate analysis of surgical type by marital status, compared to patients with non-total or non-near-total gastrectomy. SEER 2004-2012 (n=16910)

Surgery	Multivariate analysis		
	RRR	[95%CI]	P value
**No surgery**			
**Marital Status**			
Unmarried	Reference		
Married	0.75	0.69-0.81	<0.001
**Non-Total or Non-near-total gastrectomy**	(base outcome)		
**Marital Status**			
Unmarried			
Married			
**Total or near total gastrectomy**			
**Marital Status**			
Unmarried	Reference		
Married	1.03	0.93-1.14	0.55

Abbreviation: SEER=Surveillance, Epidemiology and End Results.

Supplementary Table S8: Univariate and multivariate survival analysis for marital status on overall survival (OS) by treatments. SEER 2004-2012 (n=16910)

Treatment	Univariate analysis			Multivariate analysis		
	5-year OS	Log rank χ2	P value	HR	95%CI	P value
**Surgery and radiotherapy (n=3159)**						
**Marital Status**		8.81	0.003			
Unmarried	37.59%			Reference		
Married	43.12%			0.92	0.83-1.03	0.131
**Surgery without radiotherapy (n=8108)**						
**Marital Status**		45.31	<0.001			
Unmarried	33.06%			Reference		
Married	41.24%			0.86	0.81-0.92	<0.001
**No surgery but radiotherapy (n=709)**						
**Marital Status**		0.74	0.390			
Unmarried	4.70%					
Married	4.53%					
**No surgery or radiotherapy (n=4681)**						
**Marital Status**		10.59	0.001			
Unmarried	3.11%			Reference		
Married	4.08%			0.87	0.81-0.94	<0.001
**Unknow (n=253)**						
**Marital Status**		0.19	0.665			
Unmarried	29.96%					
Married	28.48%					

Abbreviation: NOS= no other specific; SEER=Surveillance, Epidemiology and End Results.
