# Supplementary material for: The influence of marital status on the stage at diagnosis, treatment, and survival of adult patients with gastric cancer: a population-based study
Source: Oncotarget. 2016 Feb 15;8(14):22385–405. doi: 10.18632/oncotarget.7399 (PMC5410231; doi:10.18632/oncotarget.7399)
Supplement: Supplementary file 2 [file oncotarget-08-22385-s002.docx]

**Supplementary Table S1. Univariate and multivariate survival analysis for overall survival (OS) predictors. SEER 2004-2012 (n=16910)**

| **Variable** | **5-year OS** | **Univariate analysis** | | **Multivariate analysis** | | |
| --- | --- | --- | --- | --- | --- | --- |
|  |  | **Log rank χ2** | **P value** | **HR** | **95%CI** | **P value** |
| **Marital Status** |  | 121.4 | <0.001 |  |  |  |
| Unmarried | 24.61% |  |  | Reference | | |
| Married | 32.09% |  |  | 0.88 | 0.85-0.92 | <0.001 |
| **Site** |  | 499.1 | <0.001 |  |  |  |
| Fundus of stomach | 22.11% |  |  | Reference | | |
| Body of stomach | 29.06% |  |  | 0.91 | 0.82-1.01 | 0.073 |
| Gastric antrum | 33.83% |  |  | 0.89 | 0.81-0.97 | 0.011 |
| Pylorus | 31.14% |  |  | 0.95 | 0.84-1.08 | 0.411 |
| Lesser curvature of stomach, NOS | 37.87% |  |  | 0.79 | 0.71-0.88 | <0.001 |
| Greater curvature of stomach, NOS | 30.32% |  |  | 0.96 | 0.86-1.09 | 0.548 |
| Overlapping lesion of stomach | 16.51% |  |  | 1.05 | 0.95-1.16 | 0.338 |
| Stomach, NOS | 20.64% |  |  | 1.06 | 0.96-1.17 | 0.238 |
| **Sex** |  | 2.61 | 0.106 |  |  |  |
| Male | 29.87% |  |  |  |  |  |
| Female | 28.03% |  |  |  |  |  |
| **Race** |  | 226.77 | <0.001 |  |  |  |
| white | 26.48% |  |  | Reference | | |
| black | 25.80% |  |  | 1.08 | 1.02-1.14 | 0.005 |
| American Indian or Alaska Native | 24.02% |  |  | 1.16 | 0.95-1.41 | 0.135 |
| Asian or Pacific Islander | 38.65% |  |  | 0.81 | 0.77-0.86 | <0.001 |
| Unknown | 58.65% |  |  | 0.50 | 0.29-0.84 | 0.009 |
| **Age** |  | 286.76 | <0.001 |  |  |  |
| 18-27 | 14.47% |  |  | Reference | | |
| 28-37 | 27.73% |  |  | 0.79 | 0.58-1.07 | 0.128 |
| 38-57 | 32.29% |  |  | 0.83 | 0.63-1.1 | 0.191 |
| 58-69 | 33.94% |  |  | 0.96 | 0.72-1.26 | 0.757 |
| 70-84 | 27.31% |  |  | 1.35 | 1.02-1.78 | 0.036 |
| 85+ | 15.12% |  |  | 2.08 | 1.57-2.76 | <0.001 |
| **Grade** |  | 412.01 | <0.001 |  |  |  |
| Grade I (well differentiated) | 52.69% |  |  | Reference | | |
| Grade II (moderately differentiated) | 37.45% |  |  | 1.17 | 1.02-1.35 | 0.022 |
| Grade III ( poorly differentiated) | 26.29% |  |  | 1.43 | 1.25-1.64 | <0.001 |
| Grade IV (undifferentiated) | 23.97% |  |  | 1.62 | 1.34-1.96 | <0.001 |
| cell type not determined | 19.83% |  |  | 1.32 | 1.14-1.53 | <0.001 |
| **Histotype** |  | 255.64 | <0.001 |  |  |  |
| Adenocarcinoma, NOS | 28.18% |  |  | Reference | | |
| Adenocarcinoma, intestinal type | 41.38% |  |  | 0.88 | 0.82-0.94 | <0.001 |
| Carcinoma, diffuse type | 25.94% |  |  | 1.08 | 0.99-1.18 | 0.069 |
| Tubular adenocarcinoma | 38.18% |  |  | 0.98 | 0.78-1.23 | 0.842 |
| Papillary adenocarcinoma, NOS | 34.94% |  |  | 0.95 | 0.61-1.48 | 0.836 |
| Mucinous adenocarcinoma | 30.27% |  |  | 0.99 | 0.85-1.15 | 0.884 |
| Signet ring cell carcinoma | 24.09% |  |  | 1.12 | 1.06-1.18 | <0.001 |
| **TNM Stage^c^** |  | 4217.72 | <0.001 |  |  |  |
| Stage I | 53.58% |  |  | Reference | | |
| Stage II | 38.31% |  |  | 1.71 | 1.6-1.83 | <0.001 |
| Stage III | 20.13% |  |  | 3.00 | 2.8-3.21 | <0.001 |
| Stage IV | 3.42% |  |  | 3.99 | 3.74-4.26 | <0.001 |
| **Surgery** |  | 4146.02 | <0.001 |  |  |  |
| No surgery | 3.82% |  |  | Reference | | |
| Non-Total or Non-near-total gastrectomy | 40.86% |  |  | 0.36 | 0.34-0.38 | <0.001 |
| Total or near total gastrectomy | 31.25% |  |  | 0.41 | 0.39-0.44 | <0.001 |
| **Radiation** |  | 250.04 | <0.001 |  |  |  |
| No radiotherapy | 27.03% |  |  | Reference | | |
| Radiotherapy | 35.13% |  |  | 0.74 | 0.71-0.78 | <0.001 |
| Radiotherapy unknown | 28.94% |  |  | 0.86 | 0.73-1.02 | 0.076 |

Abbreviation: NOS= no other specific; SEER=Surveillance, Epidemiology and End Results;

**Supplementary Table S2. Characteristics of patients by marital status in 1:1 matching, unmarried verus married. SEER 2004-2012 (n=14056)^a^**

| **Characteristics** | **Total** | **Unmarried** | **Married** | **P value^b^** |
| --- | --- | --- | --- | --- |
|  | **14056 (100)** | **7028 (50)** | **7028 (50)** |  |
| **Sex** |  |  |  | <0.001 |
| male | 6614(47.05) | 2917(41.51) | 3697(52.6) |  |
| female | 7442(52.95) | 4111(58.49) | 3331(47.4) |  |
| **Race** |  |  |  | <0.001 |
| white | 9177(65.29) | 4155(59.12) | 5022(71.46) |  |
| black | 2430(17.29) | 1673(23.8) | 757(10.77) |  |
| American Indian/Alaska Native | 123(0.88) | 73(1.04) | 50(0.71) |  |
| Asian or Pacific Islander | 2302(16.38) | 1111(15.81) | 1191(16.95) |  |
| Unknown | 24(0.17) | 16(0.23) | 8(0.11) |  |
| **Age** |  |  |  | <0.001 |
| 18-27 | 73(0.52) | 56(0.8) | 17(0.24) |  |
| 28-37 | 324(2.31) | 179(2.55) | 145(2.06) |  |
| 38-57 | 2499(17.78) | 1379(19.62) | 1120(15.94) |  |
| 58-69 | 3578(25.46) | 1549(22.04) | 2029(28.87) |  |
| 70-84 | 5902(41.99) | 2770(39.41) | 3132(44.56) |  |
| 85+ | 1680(11.95) | 1095(15.58) | 585(8.32) |  |
| **Histotype** |  |  |  | 0.017 |
| Adenocarcinoma, NOS | 7160(50.94) | 3561(50.67) | 3599(51.21) |  |
| Adenocarcinoma, intestinal type | 2068(14.71) | 1014(14.43) | 1054(15) |  |
| Carcinoma, diffuse type | 874(6.22) | 429(6.1) | 445(6.33) |  |
| Tubular adenocarcinoma | 138(0.98) | 59(0.84) | 79(1.12) |  |
| Papillary adenocarcinoma, NOS | 32(0.23) | 18(0.26) | 14(0.2) |  |
| Mucinous adenocarcinoma | 245(1.74) | 147(2.09) | 98(1.39) |  |
| Signet ring cell carcinoma | 3539(25.18) | 1800(25.61) | 1739(24.74) |  |
| **Site** |  |  |  | 0.054 |
| Fundus of stomach | 715(5.09) | 343(4.88) | 372(5.29) |  |
| Body of stomach | 1768(12.58) | 875(12.45) | 893(12.71) |  |
| Gastric antrum | 4529(32.22) | 2295(32.66) | 2234(31.79) |  |
| Pylorus | 698(4.97) | 377(5.36) | 321(4.57) |  |
| Lesser curvature of stomach, NOS | 1774(12.62) | 891(12.68) | 883(12.56) |  |
| Greater curvature of stomach, NOS | 816(5.81) | 370(5.26) | 446(6.35) |  |
| Overlapping lesion of stomach | 1563(11.12) | 777(11.06) | 786(11.18) |  |
| Stomach, NOS | 2193(15.6) | 1100(15.65) | 1093(15.55) |  |
| **TNM Stage^c^** |  |  |  | 0.057 |
| Stage I | 3852(27.4) | 1995(28.39) | 1857(26.42) |  |
| Stage II | 3152(22.42) | 1537(21.87) | 1615(22.98) |  |
| Stage III | 3270(23.26) | 1613(22.95) | 1657(23.58) |  |
| Stage IV | 3782(26.91) | 1883(26.79) | 1899(27.02) |  |
| **Cause of Death** |  |  |  | <0.001 |
| Alive or dead of other cause | 6345(45.14) | 3068(43.65) | 3277(46.63) |  |
| Dead (attributable to this cancer dx) | 7711(54.86) | 3960(56.35) | 3751(53.37) |  |
| **Grade** |  |  |  | 0.712 |
| Grade I (well differentiated) | 519(3.69) | 265(3.77) | 254(3.61) |  |
| Grade II (moderately differentiated) | 3166(22.52) | 1604(22.82) | 1562(22.23) |  |
| Grade III ( poorly differentiated) | 8521(60.62) | 4235(60.26) | 4286(60.98) |  |
| Grade IV (undifferentiated) | 282(2.01) | 133(1.89) | 149(2.12) |  |
| Cell type not determined | 1568(11.16) | 791(11.25) | 777(11.06) |  |
| **Surgery** |  |  |  | <0.001 |
| No surgery | 4707(33.49) | 2572(36.6) | 2135(30.38) |  |
| Non-Total or Non-near-total gastrectomy | 7352(52.31) | 3540(50.37) | 3812(54.24) |  |
| Total or near total gastrectomy | 1997(14.21) | 916(13.03) | 1081(15.38) |  |
| **Radiotherapy** |  |  |  | <0.001 |
| No radiotherapy | 10856(77.23) | 5591(79.55) | 5265(74.91) |  |
| Radiotherapy | 2976(21.17) | 1337(19.02) | 1639(23.32) |  |
| Radiotherapy unknown | 224(1.59) | 100(1.42) | 124(1.76) |  |

Abbreviation: NOS= no other specific; SEER=Surveillance, Epidemiology and End Results;

^a^Data are presented as No.(percentage) of patients

^b^P values of the Chi-square test or Wilcoxon-Mann-Whitney test comparing unmarried and married groups

^c^Being restaged according to the criteria of AJCC Cancer Staging Manual (7th edition, 2010)

**Supplementary Table S3. Univariate and multivariate survival analysis for gastric cancer-caused special survival (CSS) predictors in 1:1 matching. SEER 2004-2012 (n=14056)**

| **Variable** | **5-year CSS** | **Univariate analysis** | | **Multivariate analysis** | | |
| --- | --- | --- | --- | --- | --- | --- |
|  |  | **Log rank χ2** | **P value** | **HR** | **95%CI** | **P value** |
| **Marital Status** |  | 35.35 | <0.001 |  |  |  |
| Unmarried | 32.79% |  |  | Reference | | |
| Married | 36.54% |  |  | 0.90 | 0.85-0.94 | <0.001 |
| **Site** |  | 421.52 | <0.001 |  |  |  |
| Fundus of stomach | 26.02% |  |  | Reference | | |
| Body of stomach | 33.75% |  |  | 0.94 | 0.83-1.06 | 0.311 |
| Gastric antrum | 40.87% |  |  | 0.88 | 0.79-0.98 | 0.022 |
| Pylorus | 37.64% |  |  | 0.93 | 0.80-1.08 | 0.364 |
| Lesser curvature of stomach, NOS | 43.39% |  |  | 0.80 | 0.70-0.90 | <0.001 |
| Greater curvature of stomach, NOS | 35.95% |  |  | 0.96 | 0.84-1.11 | 0.605 |
| Overlapping lesion of stomach | 21.42% |  |  | 1.05 | 0.93-1.19 | 0.396 |
| Stomach, NOS | 25.02% |  |  | 1.07 | 0.95-1.20 | 0.27 |
| **Sex** |  | 0.64 | 0.4229 |  |  |  |
| Male | 34.91% |  |  |  |  |  |
| Female | 34.54% |  |  |  |  |  |
| **Race** |  | 105.1 | <0.001 |  |  |  |
| white | 32.94% |  |  | Reference | | |
| black | 32.12% |  |  | 1.08 | 1.01-1.15 | 0.017 |
| American Indian or Alaska Native | 37.76% |  |  | 1.13 | 0.88-1.44 | 0.339 |
| Asian or Pacific Islander | 44.12% |  |  | 0.83 | 0.77-0.89 | <0.001 |
| Unknown | 75.79% |  |  | 0.20 | 0.04-0.79 | 0.022 |
| **Age** |  | 69.59 | <0.001 |  |  |  |
| 18-27 | 19.91% |  |  | Reference | | |
| 28-37 | 31.87% |  |  | 0.74 | 0.52-1.03 | 0.077 |
| 38-57 | 32.94% |  |  | 0.85 | 0.63-1.16 | 0.304 |
| 58-69 | 38.30% |  |  | 0.97 | 0.71-1.32 | 0.857 |
| 70-84 | 35.33% |  |  | 1.28 | 0.94-1.73 | 0.12 |
| 85+ | 29.08% |  |  | 1.85 | 1.36-2.53 | <0.001 |
| **Grade** |  | 434.97 | <0.001 |  |  |  |
| Grade I (well differentiated) | 67.52% |  |  | Reference | | |
| Grade II (moderately differentiated) | 45.96% |  |  | 1.35 | 1.12-1.62 | 0.002 |
| Grade III ( poorly differentiated) | 30.46% |  |  | 1.76 | 1.47-2.11 | <0.001 |
| Grade IV (undifferentiated) | 24.74% |  |  | 2.14 | 1.69-2.71 | <0.001 |
| cell type not determined | 25.37% |  |  | 1.57 | 1.29-1.90 | <0.001 |
| **Histotype** |  | 294.9 | <0.001 |  |  |  |
| Adenocarcinoma, NOS | 34.16% |  |  | Reference | | |
| Adenocarcinoma, intestinal type | 50.97% |  |  | 0.83 | 0.77-0.90 | <0.001 |
| Carcinoma, diffuse type | 30.36% |  |  | 1.07 | 0.97-1.19 | 0.193 |
| Tubular adenocarcinoma | 44.98% |  |  | 1.02 | 0.77-1.33 | 0.909 |
| Papillary adenocarcinoma, NOS | 43.76% |  |  | 0.92 | 0.51-1.67 | 0.79 |
| Mucinous adenocarcinoma | 37.88% |  |  | 0.94 | 0.78-1.13 | 0.528 |
| Signet ring cell carcinoma | 26.58% |  |  | 1.12 | 1.06-1.19 | <0.001 |
| **TNM Stage^c^** |  | 3732.67 | <0.001 |  |  |  |
| Stage I | 63.97% |  |  | Reference | | |
| Stage II | 44.78% |  |  | 1.95 | 1.79-2.12 | <0.001 |
| Stage III | 22.74% |  |  | 3.58 | 3.30-3.89 | <0.001 |
| Stage IV | 3.68% |  |  | 4.68 | 4.32-5.07 | <0.001 |
| **Surgery** |  | 3265.54 | <0.001 |  |  |  |
| No surgery | 6.16% |  |  | Reference | | |
| Non-Total or Non-near-total gastrectomy | 48.18% |  |  | 0.34 | 0.32-0.36 | <0.001 |
| Total or near total gastrectomy | 35.96% |  |  | 0.39 | 0.36-0.42 | <0.001 |
| **Radiation** |  | 127.23 | <0.001 |  |  |  |
| No radiotherapy | 33.49% |  |  | Reference | | |
| Radiotherapy | 38.70% |  |  | 0.76 | 0.71-0.81 | <0.001 |
| Radiotherapy unknown | 37.29% |  |  | 0.87 | 0.71-1.06 | 0.157 |

Abbreviation: NOS= no other specific; SEER=Surveillance, Epidemiology and End Results;

**Supplementary Table S5. Characteristics of patients by surgery with corresponding multinomia univariate analysis. SEER 2004-2012 (n=16910)^a^**

| **Characteristics** | **Total** | **No surgery** | **Non-Total or Non-near-total gastrectomy** | **Total or near total gastrectomy** | **Univariate analysis** |
| --- | --- | --- | --- | --- | --- |
|  | **16910 (100)** | **5438 (100)** | **8953 (100)** | **2519 (100)** | **P value^b^** |
| **Marital Status** |  |  |  |  | <0.001 |
| Unmarried | 7028 (41.56) | 2572 (47.3) | 3540 (39.54) | 916 (36.36) |  |
| Married | 9882 (58.44) | 2866 (52.7) | 5413 (60.46) | 1603 (63.64) |  |
| **Site** |  |  |  |  | <0.001 |
| Fundus of stomach | 857 (5.07) | 380 (6.99) | 259 (2.89) | 218 (8.65) |  |
| Body of stomach | 2115 (12.51) | 754 (13.87) | 976 (10.9) | 385 (15.28) |  |
| Gastric antrum | 5440 (32.17) | 1376 (25.3) | 3688 (41.19) | 376 (14.93) |  |
| Pylorus | 839 (4.96) | 170 (3.13) | 639 (7.14) | 30 (1.19) |  |
| Lesser curvature of stomach, NOS | 2207 (13.05) | 468 (8.61) | 1229 (13.73) | 510 (20.25) |  |
| Greater curvature of stomach, NOS | 990 (5.85) | 256 (4.71) | 581 (6.49) | 153 (6.07) |  |
| Overlapping lesion of stomach | 1892 (11.19) | 768 (14.12) | 693 (7.74) | 431 (17.11) |  |
| Stomach, NOS | 2570 (15.2) | 1266 (23.28) | 888 (9.92) | 416 (16.51) |  |
| **Sex** |  |  |  |  | 0.834 |
| Male | 9463 (55.96) | 3025 (55.63) | 5024 (56.12) | 1414 (56.13) |  |
| Female | 7447 (44.04) | 2413 (44.37) | 3929 (43.88) | 1105 (43.87) |  |
| **Race** |  |  |  |  | <0.001 |
| white | 10298 (60.9) | 3497 (64.31) | 5185 (57.91) | 1616 (64.15) |  |
| black | 2804 (16.58) | 1008 (18.54) | 1451 (16.21) | 345 (13.7) |  |
| American Indian or Alaska Native | 159 (0.94) | 61 (1.12) | 64 (0.71) | 34 (1.35) |  |
| Asian or Pacific Islander | 853 (5.04) | 853 (15.69) | 853 (9.53) | 853 (33.86) |  |
| Unknown | 56 (0.33) | 19 (0.35) | 27 (0.3) | 10 (0.4) |  |
| **Age** |  |  |  |  | <0.001 |
| 18-27 | 84 (0.5) | 45 (0.83) | 18 (0.2) | 21 (0.83) |  |
| 28-37 | 437 (2.58) | 193 (3.55) | 168 (1.88) | 76 (3.02) |  |
| 38-57 | 3877 (22.93) | 1248 (22.95) | 1897 (21.19) | 732 (29.06) |  |
| 58-69 | 4442 (26.27) | 1295 (23.81) | 2384 (26.63) | 763 (30.29) |  |
| 70-84 | 6376 (37.71) | 1884 (34.65) | 3674 (41.04) | 818 (32.47) |  |
| 85+ | 1694 (10.02) | 773 (14.21) | 812 (9.07) | 109 (4.33) |  |
| **Grade** |  |  |  |  | <0.001 |
| Grade I (well differentiated) | 598 (3.54) | 117 (2.15) | 429 (4.79) | 52 (2.06) |  |
| Grade II (moderately differentiated) | 3696 (21.86) | 983 (18.08) | 2279 (25.46) | 434 (17.23) |  |
| Grade III ( poorly differentiated) | 10534 (62.29) | 3077 (56.58) | 5613 (62.69) | 1844 (73.2) |  |
| Grade IV (undifferentiated) | 340 (2.01) | 65 (1.2) | 194 (2.17) | 81 (3.22) |  |
| cell type not determined | 1742 (10.3) | 1196 (21.99) | 438 (4.89) | 108 (4.29) |  |
| **Histotype** |  |  |  |  | <0.001 |
| Adenocarcinoma, NOS | 8279 (48.96) | 3150 (57.93) | 4182 (46.71) | 947 (37.59) |  |
| Adenocarcinoma, intestinal type | 2435 (14.4) | 363 (6.68) | 1718 (19.19) | 354 (14.05) |  |
| Carcinoma, diffuse type | 1084 (6.41) | 262 (4.82) | 566 (6.32) | 256 (10.16) |  |
| Tubular adenocarcinoma | 166 (0.98) | 16 (0.29) | 125 (1.4) | 25 (0.99) |  |
| Papillary adenocarcinoma, NOS | 41 (0.24) | 5 (0.09) | 29 (0.32) | 7 (0.28) |  |
| Mucinous adenocarcinoma | 311 (1.84) | 60 (1.1) | 204 (2.28) | 47 (1.87) |  |
| Signet ring cell carcinoma | 4594 (27.17) | 1582 (29.09) | 2129 (23.78) | 883 (35.05) |  |
| **TNM Stage^c^** |  |  |  |  | <0.001 |
| Stage I | 4468 (26.42) | 1368 (25.16) | 2684 (29.98) | 416 (16.51) |  |
| Stage II | 3724 (22.02) | 652 (11.99) | 2467 (27.56) | 605 (24.02) |  |
| Stage III | 4077 (24.11) | 344 (6.33) | 2647 (29.57) | 1086 (43.11) |  |
| Stage IV | 4641 (27.45) | 3074 (56.53) | 1155 (12.9) | 412 (16.36) |  |

Abbreviation: NOS= no other specific; SEER=Surveillance, Epidemiology and End Results;

^a^Data are presented as No.(percentage) of patients.

^b^P values are from multinomia univariate logistic tests.

^c^Being restaged according to the criteria of AJCC Cancer Staging Manual (7th edition, 2010)

**Supplementary Table S6. Characteristics of patients by radiotherapy with corresponding multinomia univariate analysis. SEER 2004-2012 (n=16910)^a^**

| **Characteristics** | **Total** | **No radiotherapy** | **Radiotherapy** | **Radiotherapy unknown** | **Univariate analysis** |
| --- | --- | --- | --- | --- | --- |
|  | **16910 (100)** | **12789 (100)** | **3868 (100)** | **253 (100)** | **P value^b^** |
| **Marital Status** |  |  |  |  | <0.001 |
| Unmarried | 7028 (41.56) | 5591 (43.72) | 1337 (34.57) | 100 (39.53) |  |
| Married | 9882 (58.44) | 7198 (56.28) | 2531 (65.43) | 153 (60.47) |  |
| **Site** |  |  |  |  | <0.001 |
| Fundus of stomach | 857 (5.07) | 634 (4.96) | 215 (5.56) | 8 (3.16) |  |
| Body of stomach | 2115 (12.51) | 1633 (12.77) | 445 (11.5) | 37 (14.62) |  |
| Gastric antrum | 5440 (32.17) | 4094 (32.01) | 1267 (32.76) | 79 (31.23) |  |
| Pylorus | 839 (4.96) | 599 (4.68) | 226 (5.84) | 14 (5.53) |  |
| Lesser curvature of stomach, NOS | 2207 (13.05) | 1598 (12.5) | 580 (14.99) | 29 (11.46) |  |
| Greater curvature of stomach, NOS | 990 (5.85) | 734 (5.74) | 244 (6.31) | 12 (4.74) |  |
| Overlapping lesion of stomach | 1892 (11.19) | 1414 (11.06) | 444 (11.48) | 34 (13.44) |  |
| Stomach, NOS | 2570 (15.2) | 2083 (16.29) | 447 (11.56) | 40 (15.81) |  |
| **Sex** |  |  |  |  | <0.001 |
| Male | 9463 (55.96) | 7041 (55.06) | 2271 (58.71) | 151 (59.68) |  |
| Female | 7447 (44.04) | 5748 (44.94) | 1597 (41.29) | 102 (40.32) |  |
| **Race** |  |  |  |  | <0.001 |
| white | 10298 (60.9) | 7879 (61.61) | 2244 (58.01) | 175 (69.17) |  |
| black | 2804 (16.58) | 2102 (16.44) | 670 (17.32) | 32 (12.65) |  |
| American Indian or Alaska Native | 159 (0.94) | 124 (0.97) | 32 (0.83) | 3 (1.19) |  |
| Asian or Pacific Islander | 3593 (21.25) | 2640 (20.64) | 912 (23.58) | 41 (16.21) |  |
| Unknown | 56 (0.33) | 44 (0.34) | 10 (0.26) | 2 (0.79) |  |
| **Age** |  |  |  |  | <0.001 |
| 18-27 | 84 (0.5) | 63 (0.49) | 17 (0.44) | 4 (1.58) |  |
| 28-37 | 437 (2.58) | 300 (2.35) | 129 (3.34) | 8 (3.16) |  |
| 38-57 | 3877 (22.93) | 2598 (20.31) | 1218 (31.49) | 61 (24.11) |  |
| 58-69 | 4442 (26.27) | 3137 (24.53) | 1225 (31.67) | 80 (31.62) |  |
| 70-84 | 6376 (37.71) | 5163 (40.37) | 1130 (29.21) | 83 (32.81) |  |
| 85+ | 1694 (10.02) | 1528 (11.95) | 149 (3.85) | 17 (6.72) |  |
| **Grade** |  |  |  |  | <0.001 |
| Grade I (well differentiated) | 598 (3.54) | 522 (4.08) | 69 (1.78) | 7 (2.77) |  |
| Grade II (moderately differentiated) | 3696 (21.86) | 2900 (22.68) | 738 (19.08) | 58 (22.92) |  |
| Grade III ( poorly differentiated) | 10534 (62.29) | 7645 (59.78) | 2719 (70.29) | 170 (67.19) |  |
| Grade IV (undifferentiated) | 340 (2.01) | 237 (1.85) | 98 (2.53) | 5 (1.98) |  |
| cell type not determined | 1742 (10.3) | 1485 (11.61) | 244 (6.31) | 13 (5.14) |  |
| **Histotype** |  |  |  |  | <0.001 |
| Adenocarcinoma, NOS | 8279 (48.96) | 6424 (50.23) | 1731 (44.75) | 124 (49.01) |  |
| Adenocarcinoma, intestinal type | 2435 (14.4) | 1873 (14.65) | 532 (13.75) | 30 (11.86) |  |
| Carcinoma, diffuse type | 1084 (6.41) | 741 (5.79) | 327 (8.45) | 16 (6.32) |  |
| Tubular adenocarcinoma | 166 (0.98) | 141 (1.1) | 23 (0.59) | 2 (0.79) |  |
| Papillary adenocarcinoma, NOS | 41 (0.24) | 31 (0.24) | 10 (0.26) | 0 (0) |  |
| Mucinous adenocarcinoma | 311 (1.84) | 220 (1.72) | 85 (2.2) | 6 (2.37) |  |
| Signet ring cell carcinoma | 4594 (27.17) | 3359 (26.26) | 1160 (29.99) | 75 (29.64) |  |
| **TNM Stage^c^** |  |  |  |  | <0.001 |
| Stage I | 4468 (26.42) | 4012 (31.37) | 411 (10.63) | 45 (17.79) |  |
| Stage II | 3724 (22.02) | 2398 (18.75) | 1258 (32.52) | 68 (26.88) |  |
| Stage III | 4077 (24.11) | 2325 (18.18) | 1662 (42.97) | 90 (35.57) |  |
| Stage IV | 4641 (27.45) | 4054 (31.7) | 537 (13.88) | 50 (19.76) |  |

Abbreviation: NOS= no other specific; SEER=Surveillance, Epidemiology and End Results;

^a^Data are presented as No.(percentage) of patients.

^b^P values are from multinomia univariate logistic tests.

^c^Being restaged according to the criteria of AJCC Cancer Staging Manual (7th edition, 2010)
